# Temporal localization of optical waves supported by a copropagating quasiperiodic structure

**DOI:** 10.1515/nanoph-2024-0571

**Published:** 2025-01-08

**Authors:** Majid Yazdani-Kachoei, Krzysztof Sacha, Boris A. Malomed

**Affiliations:** Instytut Fizyki Teoretycznej, Wydział Fizyki, Astronomii i Informatyki Stosowanej, Uniwersytet Jagielloński, ul. Profesora Stanisława Łojasiewicza 11, PL-30-348 Kraków, Poland; Centrum Marka Kaca, Uniwersytet Jagielloński, ulica Profesora Stanisława Łojasiewicza 11, PL-30-348 Kraków, Poland; Department of Physical Electronics, School of Electrical Engineering, Faculty of Engineering, Tel Aviv University, Tel Aviv 69978, Israel; Instituto de Alta Investigación, Universidad de Tarapacá, Casilla 7D, Arica, Chile

**Keywords:** time crystals, temporal phenomena, localization

## Abstract

Research on time crystals concerns the spontaneous breaking of translational symmetry in time, as well as the realization of phenomena and phases known from solid-state physics in the time domain. Periodically driven systems of massive particles are widely used in these studies. In the present work, we consider a photonic system and demonstrate that stable nonlinear propagation of a strong optical wave in a fiber with the third-order dispersion may lead to the establishment of quasi-periodic oscillations in the electromagnetic field intensity. A second, weaker signal optical wave propagating in the fiber senses these oscillations and, as a result, undergoes exponential localization in time. This is a temporal analog of Aubry–André localization. If an optical detector is placed at a certain position in the fiber, the temporal localization of the probe wave will be observed in the form of the signal which emerges and then decays as a function of time.

## 1 Introduction

A quantum particle in the presence of a stationary potential with a disordered spatial structure may exhibit Anderson localization (AL), which gives rise to eigenstates exponentially localized at different positions in space [1], [2]. AL was studied in a broad range of phenomenology modeled by disordered wave equations, ranging from the sound propagation to matter waves [3]. In particular, this effect was predicted and observed in the propagation of light [4], [5] and plasmonic [6] excitations in random optical and photonic media [7], [8], [9]. Typically, AL is observed in the configuration space, resulting from the presence of time-independent spatial disorder. It can also be observed in the momentum space of a quantum particle. In that case, it is associated with the quantum suppression of classical diffusion in systems that are classically chaotic [10], [11].

Recently, it has been shown that AL is also possible in the time domain [12], [13], [14], [15]. This phenomenon belongs to the field of time crystals, which has been intensively elaborated over the past decade [15], [16], [17], [18]. Akin to their spatial counterparts, time crystals exhibit spontaneous regular self-organization – not in space, but in the time domain [19], [20], [21], [22], [23], [24], [25], [26], [27], [28], [29], [30], [31], [32], [33], [34], [35], [36], [37], [38], [39], [40], [41], [42], [43], [44], [45], [46]. This self-organization involves spontaneous breaking of the time-translation symmetry, thus being a temporal counterpart of one of the most important features of solid-state physics. In time-crystal setups, it is also possible to realize a wide range of phases and phenomena known in condensed matter physics, which are not necessarily associated with the spontaneous breaking of the time-translation symmetry [15], [17], [47], [48], [49]. These include, in particular, AL in the time domain [12], [13], [14], [15]: a quantum particle perturbed by a fluctuating external force reveals localization in time, meaning that, if one places a detector at a certain spatial position, temporal variations of the detection probability exhibit exponential localization around a certain moment in time. The description of this phenomenon reduces to the Anderson model.

Temporal disorder has also been investigated in photonic time crystals. These are spatially homogeneous dielectric materials where the refractive index is periodically modulated in time. Solutions of the respective Maxwell equations reveal band structures in the momentum space [50], [51], [52]. Essentially, the temporal disorder leads to an unbounded amplification of electromagnetic pulses over time and slowdown of their propagation through the material [53], [54], [55], [56], [57].

In the three-dimensional (3D) space, AL is present if the underlying disorder is sufficiently strong – namely, 3D eigenstates with a given energy localize if the disorder strength exceeds a critical value [58]. In 2D and 1D cases, even arbitrarily weak disorder leads to AL, although in 2D the localization length of the wave function of the quantum particle may be very large. On the other hand, in the 1D case, replacing the random spatial potential by a lattice (periodic) one, which is subject to superlattice modulation with an incommensurate spatial period, one arrives at the Aubry–André (AA) model with an effective *quasiperiodic* (QP) potential [59]. In spite of the absence of disorder in the AA model, sufficiently strong modulation also leads to the localization of eigenstates of the quantum particle.

In its strict sense, the AA model is a tight-binding discrete one characterized by rate of tunneling between sites of a 1D chain and on-site energies that vary in a QP manner along the chain. For fixed on-site energies, the localization of eigenstates takes place if the tunneling rate falls below a certain critical value, i.e., the delocalizing overlap between adjacent sites is not too strong [59]. While our system is continuous, rather than discrete one, we demonstrate that the localization mechanism acting in the system is quite similar to its counterpart in the AA model.

Fiber optics offers a specific possibility to create an appropriate setting for the localization in the temporal domain, instead of the spatial one. Namely, one can consider copropagation of two waves carried by two different channels in the same fiber, which represent different wavelengths. In the *support channel*, the wave is assumed to be strong; hence, it propagates in the nonlinear regime, with the corresponding carrier wavelength belonging to the range of normal group-velocity dispersion (GVD) [60]. Then, this wave can develop a stable periodically modulated shape. Roughly speaking, it is a chain of dark solitons, alias a *cnoidal* wave, so called because it is represented by an exact solution of the corresponding nonlinear Schrödinger (NLS) equation expressed in terms of Jacobi elliptic functions, such as cn [61]. Through the cross-phase-modulation (XPM) effect, the cnoidal wave induces an effective periodic potential in the second, *signal channel*, which carries optical signals. In particular, in Refs. [62] and [63] the situation was considered when the signal channel operated with anomalous GVD, thus carrying signals in the form of bright solitons (a similar, but less stable, scheme with a chain of bright solitons created in the support channel with anomalous GVD was recently considered in Ref. [64]). A well-known problem in fiber-optic telecommunications is the temporal jitter, alias the Gordon–Haus effect [65], i.e., random walk of the solitons in the temporal domain, caused by their interaction with random optical noise spontaneously emitted by amplifiers, which must be periodically inserted in the long fiber link for the compensation of losses. Although the jitter does not destroy solitons, it tends to obliterate data encoded by temporal positions of the solitons in the signal stream. As proposed in Refs. [62] and [63], the effective XPM-induced periodic (cnoidal-like) potential, acting on the solitons in the signal channel, may effectively suppress the jitter, thus stabilizing the data transmission by the soliton stream.

The two-channel support-signal scheme, which can be implemented in the optical fiber, suggests a possibility to induce an effective QP (rather than periodic) potential in the signal channel. To this end, the carrier wavelength in the support channel should be taken close enough to the zero-dispersion point, which makes it necessary to take into regard the third-order GVD, in addition to the usual second-order normal-GVD term [60]. Indeed, the NLS equation that includes solely the second-order GVD is commonly known to be integrable [66]. This fact makes the effective second-order ordinary differential equation (ODE) for the wave amplitude in the support channel also integrable. As a result, the integrable ODE, being formally equivalent to the mechanical equation of motion for a particle in an external potential well, is integrable too, producing only periodic solutions of the cnoidal type (the soliton is a limit case of the solution with an infinite period). On the other hand, the addition of the terms accounting for the third-order GVD breaks the integrability of the NLS equation and makes its ODE reduction for stationary waves also nonintegrable. As we demonstrate below, the stationary waves exist, in the support channel, as robust solutions with a QP structure. Then, by dint of the XPM effect, they induce an effective QP potential in the signal channel. We consider the wave in the signal channel as a weak one, governed by the linear propagation equation. Under the action of the XPM-induced QP potential, the signal wave develops the temporal-domain localization, similar to that in the spatial-domain AA model.

The subsequent presentation is organized as follows: the two-channel model is introduced in Section 2, where we also formulate the framework for the analysis of the QP solutions in terms of the single NLS equation, which represents the support channel. The equation includes the second- and third-order GVD terms (with the positive second-order GVD coefficient, which corresponds to the normal GVD, as, in the case of the anomalous GVD, the QP waves may be subject to the modulational instability) and the cubic Kerr nonlinearity. The complex ODE that produces QP waves in the support channel is tantamount to a real dynamical system with a six-dimensional phase space. We derive two dynamical invariants of the six-dimensional system, *viz*., its Hamiltonian and a conserved quantity corresponding to the phase invariance of the complex ODE. The situation seems as the one for a system with three degrees of freedom and two dynamical invariants, which implies that the system is nonintegrable. Therefore, it is able to produce QP solutions. In Section 3, numerical solutions are produced: first, QP waves in the support channel, then solutions of the linear Schrödinger equation in the signal channel, with the effective XPM-induced QP potential. The latter solutions demonstrate the localization of the signal wave function in the time domain, which is the main result reported by the present work. The paper is concluded by Section 4.

## 2 The model and support structure: analytical considerations

The system of NLS equations for the copropagation of optical waves with envelope amplitudes *U*(*z*, *t*) and Ψ(*z*, *t*) in the support and signal channels, respectively, carried by different wavelengths in the fiber, is written as [60], [62], [63]

(1) $$i\frac{\partial U}{\partial z}=\left[ic\frac{\partial }{\partial t}+\frac{\beta_{2}}{2}\frac{\partial^{2}}{\partial t^{2}}+\frac{i\beta_{3}}{6}\frac{\partial^{3}}{\partial t^{3}}-|U\left(z,t\right){|}^{2}\right]U,$$

(2) $$i\frac{\partial \Psi }{\partial z}=\left[\frac{\gamma_{2}}{2}\frac{\partial^{2}}{\partial t^{2}}+\frac{i\gamma_{3}}{6}\frac{\partial^{3}}{\partial t^{3}}-2|U\left(z,t\right){|}^{2}\right]\Psi ,$$

where *z* is the propagation distance, *t* ≡ *τ* − *z*/*V*_gr_ is the reduced (local) time, defined for the signal channel which is carried by the electromagnetic wave with group velocity *V*_gr_ (*τ* is time per se), real *c* is the group-velocity mismatch between the channels, while *β*_2,3_ and *γ*_2,3_ are real coefficients of the second- and third-order GVD in the support and signal channels, respectively. Recall that the cases of *β*_2_, *γ*_2_ > 0 and <0 are classified, respectively, as normal or anomalous second-order GVD [60]. The cubic term in Eq. (1) represents the usual SPM (self-phase-modulation) nonlinearity acting in the support channel, and the last term in Eq. (2) accounts for the action of XPM in the signal channel.

In Refs. [62] and [63], the carrier wavelengths of the support and signal channels were chosen symmetrically with respect to the zero-dispersion point, which implies *β*_2_ = −*γ*_2_ and *c* = 0. In the case of small *c* ≠ 0, the respective term can be eliminated from Eq. (1) by means of the substitution that does not affect Eq. (2), *viz*., $U\left(z,t\right)\equiv \tilde{U}\left(z,t\right)\exp\left[-\left(c^{2}/\beta_{2}\right)z-i\left(c/\beta_{2}\right)t\right]$ , $\beta_{2}\to \beta_{2}+\left(\beta_{3}/\beta_{2}\right)c$ . Therefore, we drop the term *ic*∂*U*/∂*t* in Eq. (1).

As said above, the nonlinear (XPM and SPM) effects produced by the weak wave on the support and signal channels are neglected in Eqs. (1) and (2). The coefficients accounting for the SPM and XPM effects (with the usual ratio, XPM/SPM = 2 [60]) of the strong wave on the support and signal channels are set to be 1 and 2, respectively, by means of rescaling.

Because Eq. (1) does not couple to Eq. (2), the former equation can be solved separately. Once the solution is known, we proceed to solving the latter equation, where |*U*(*z*, *t*)|^2^ acts as an external potential. Aiming to realize the AA localization in the temporal domain, we are interested in a QP effective potential. To investigate this possibility, stationary solutions to Eq. (1) with an arbitrary real propagation constant *k* are looked for as

(3) $$U\left(z,t\right)=e^{i\mathrm{kz}}u\left(t\right),$$

where complex function *u*(*t*) satisfies the third-order ODE:

(4) $$ku+\frac{1}{2}\beta_{2}\frac{d^{2}u}{dt^{2}}+\frac{i\beta_{3}}{6}\frac{d^{3}u}{dt^{3}}-|u{|}^{2}u=0,$$

which is tantamount to a real dynamical system in the six-dimensional phase space, if the complex variable *u*(*t*) is split into the real and imaginary parts, *a*_1_ and *a*_2_:

(5) $$u\left(t\right)\equiv a_{1}\left(t\right)+ia_{2}\left(t\right).$$

To conclude what kind of solutions may be expected (periodic, quasiperiodic, random, or unbounded), it is crucially important to identify dynamical invariants (conserved quantities) of Eq. (4).

First, to identify the dynamical invariant, which is related, by way of the Noether theorem [67], to the invariance of Eq. (4) with respect to an arbitrary phase shift of the complex function *u*(*t*), we note that Eq. (4) can be derived from the corresponding real Lagrangian:

(6) $$\begin{array}{ll} L & =k|u{|}^{2}-\frac{1}{2}\beta_{2}{\left|\frac{du}{dt}\right|}^{2}\\ & \;+\frac{i\beta_{3}}{12}\left[{\left(\frac{d^{2}u}{dt^{2}}\right)}^{*}\frac{du}{dt}-\frac{d^{2}u}{dt^{2}}{\left(\frac{du}{dt}\right)}^{*}\right]-\frac{1}{2}|u{|}^{4}, \end{array}$$

where ∗ stands for the complex conjugate. Further, the Lagrangian can be written in terms of the Madelung substitution,

(7) $$u\left(t\right)=A\left(t\right)\exp\left(i\phi \left(t\right)\right),$$

with real amplitude $A\left(t\right)\equiv \left|u\left(t\right)\right|$ and phase *ϕ*(*t*). The result is

(8) $$\begin{array}{ll} L & =kA^{2}-\frac{1}{2}A^{4}-\frac{1}{2}\beta_{2}\left[{\left(\frac{dA}{dt}\right)}^{2}+A^{2}{\left(\frac{d\phi }{dt}\right)}^{2}\right]\\ & \;+\frac{\beta_{3}}{6}\left[2{\left(\frac{dA}{dt}\right)}^{2}\frac{d\phi }{dt}-A\frac{d^{2}A}{dt^{2}}\frac{d\phi }{dt}\right.\\ & \;\left.+A^{2}{\left(\frac{d\phi }{dt}\right)}^{3}+A\frac{dA}{dt}\frac{d^{2}\phi }{dt^{2}}\right]. \end{array}$$

Lagrangian (8) can be further transformed by replacing the last term by the one produced by the integration by parts, if one considers the corresponding integral for the action, ∫Ldt , *viz*.,

(9) $$A\frac{dA}{dt}\frac{d^{2}\phi }{dt^{2}}\to -\frac{d}{dt}\left(A\frac{dA}{dt}\right)\frac{d\phi }{dt},$$

where we further substitute

(10) $$\frac{d}{dt}\left(A\frac{dA}{dt}\right)\frac{d\phi }{dt}\equiv \left[{\left(\frac{dA}{dt}\right)}^{2}+A\frac{d^{2}A}{dt^{2}}\right]\frac{d\phi }{dt}.$$

The respectively transformed Lagrangian is

(11) $$\begin{array}{ll} \bar{L} & =kA^{2}-\frac{1}{2}A^{4}-\frac{1}{2}\beta_{2}\left[{\left(\frac{dA}{dt}\right)}^{2}+A^{2}{\left(\frac{d\phi }{dt}\right)}^{2}\right]\\ & \;+\frac{\beta_{3}}{6}\left[{\left(\frac{dA}{dt}\right)}^{2}\frac{d\phi }{dt}-2A\frac{d^{2}A}{dt^{2}}\frac{d\phi }{dt}+A^{2}{\left(\frac{d\phi }{dt}\right)}^{3}\right]. \end{array}$$

Finally, the standard variational procedure, applied to Lagrangian (11), demonstrates that the dynamical invariant sought for is

(12) $$\begin{array}{ll} I & \equiv \frac{\partial \bar{L}}{\partial \left(d\phi /dt\right)}=-\beta_{2}A^{2}\frac{d\phi }{dt}\\ & \;+\frac{\beta_{3}}{6}\left[{\left(\frac{dA}{dt}\right)}^{2}-2A\frac{d^{2}A}{dt^{2}}+3A^{2}{\left(\frac{d\phi }{dt}\right)}^{2}\right]. \end{array}$$

In the case of *β*_3_ = 0, this dynamical invariant is a commonly known one (the angular momentum in the plane of coordinates $\left(a_{1},a_{2}\right)$ , see Eq. (5)), while, to the best of our knowledge, it was not previously reported in the case of *β*_3_ ≠ 0.

The second dynamical invariant of Eq. (4) is its Hamiltonian. To derive it, one can use the representation of the solution in the form of Eq. (5), instead of the Madelung form (7). The substitution of this in the underlying expression (6) yields the Lagrangian in another form:

(13) $$\begin{array}{ll} L & =k\left(a_{1}^{2}+a_{2}^{2}\right)-\frac{1}{2}{\left(a_{1}^{2}+a_{2}^{2}\right)}^{2}-\frac{1}{2}\beta_{2}\left[{\left(\frac{da_{1}}{dt}\right)}^{2}+{\left(\frac{da_{2}}{dt}\right)}^{2}\right]\\ & \;+\frac{\beta_{3}}{6}\left(\frac{da_{1}}{dt}\frac{d^{2}a_{2}}{dt^{2}}-\frac{da_{2}}{dt}\frac{d^{2}a_{1}}{dt^{2}}\right). \end{array}$$

To derive the Hamiltonian from Lagrangian (13), it is necessary to define momentum-like variables,

(14) $$b_{1,2}\equiv \frac{da_{1,2}}{dt},$$

in addition to coordinates *a*_1,2_. Then, it is easy to check that the correct equations for *a*_1,2_, together with relation (14), can be derived from the following modification of the Lagrangian, written in terms of both *a*_1,2_ and *b*_1,2_:

(15) $$\begin{array}{ll} L_{ab} & =k\left(a_{1}^{2}+a_{2}^{2}\right)-\frac{1}{2}{\left(a_{1}^{2}+a_{2}^{2}\right)}^{2}+\frac{1}{2}\beta_{2}\left(b_{1}^{2}+b_{2}^{2}\right)\\ & \;-\beta_{2}\left(\frac{da_{1}}{dt}b_{1}+\frac{da_{2}}{dt}b_{2}\right)-\frac{\beta_{3}}{12}\left(b_{1}\frac{db_{2}}{dt}-b_{2}\frac{db_{1}}{dt}\right)\\ & \;+\frac{\beta_{3}}{6}\left(\frac{da_{1}}{dt}\frac{db_{2}}{dt}-\frac{da_{2}}{dt}\frac{db_{1}}{dt}\right). \end{array}$$

Finally, the canonical form of Lagrangian (15), which includes only the first derivatives, makes it possible to construct the conserved Hamiltonian by means of the Legendre transformation [67]:

(16) $$\begin{array}{ll} H & =\overset{2}{\underset{j=1}{\sum }}\left[\frac{\partial L_{ab}}{\partial \left(da_{j}/dt\right)}\frac{da_{j}}{dt}+\frac{\partial L_{ab}}{\partial \left(db_{j}/dt\right)}\frac{db_{j}}{dt}\right]-L_{ab}\\ & =-k\left(a_{1}^{2}+a_{2}^{2}\right)+\frac{1}{2}{\left(a_{1}^{2}+a_{2}^{2}\right)}^{2}-\frac{1}{2}\beta_{2}\left(b_{1}^{2}+b_{2}^{2}\right)\\ & \;+\frac{\beta_{3}}{6}\left(\frac{da_{1}}{dt}\frac{db_{2}}{dt}-\frac{da_{2}}{dt}\frac{db_{1}}{dt}\right). \end{array}$$

This expression for the Hamiltonian of Eq. (4) with *β*_3_ ≠ 0 has not been reported previously either, to the best of our knowledge.

Thus, the dynamical system in the six-dimensional phase space, which corresponds to Eq. (4), maintains two dynamical invariants, given by expressions (12) and (16). For this reason, as mentioned above, the system *is not integrable* (on the contrary to its commonly known four-dimensional limit with *β*_3_ = 0); hence, its bounded generic solutions should be quasiperiodic and/or chaotic.

## 3 Numerical results

### 3.1 Quasi-periodic waves in the support channel

In the case of *β*_3_ ≠ 0, analytical solutions to Eq. (4) are not available. The key goal of the numerical solution of this equation was to produce the “most irregular” (nonperiodic) evolution of |*u*(*t*)|^2^ that remains bounded (does not develop singularities).

Before proceeding to numerical results, we start with an analysis of initial conditions in the case of *β*_3_ = 0, i.e., when Eq. (4) is integrable. In terms of variables *x* ≡ *a*_1_, *y* ≡ *a*_2_ and *p*_*x*_ ≡ *b*_1_, *p*_*y*_ ≡ *b*_2_, Hamiltonian (16) with *β*_3_ = 0 is

(17) $$H=-\left[\beta_{2}\frac{p_{x}^{2}+p_{y}^{2}}{2}+k\left(x^{2}+y^{2}\right)-\frac{{\left(x^{2}+y^{2}\right)}^{2}}{2}\right],$$

which is tantamount to the Hamiltonian of a particle moving in the 2D plane $\left(x,y\right)$ under the action of a rotationally symmetric potential. The angular momentum of such a particle, *L* = *xp*_*y*_ − *yp*_*x*_ is conserved (*L* ≡ − *I*/*β*_2_, where *I* is dynamical invariant (12) in the case of *β*_3_ = 0). Consequently, the time evolution of |*u*(*t*)|^2^ ≡ *x* ^2^(*t*) + *y* ^2^(*t*), which determines the external potential for the signal channel, can be either periodic or unbounded (as mentioned above), unbounded solutions being possible for *β*_2_ > 0. In what follows below, while numerically integrating Eq. (4) with *β*_2_ > 0, we chose initial conditions so that the motion would be bounded for *β*_3_ = 0.

It might seem, formally, that choosing the anomalous-GVD sign, *β*_2_ < 0, in the support channel, which gives rise to the classical bright solitons [60], [66] and ensures the boundedness of solutions for *β*_3_ = 0, might be the best choice, but this is not the case. When *β*_3_ ≠ 0, our numerical results demonstrate that the maximum value of *β*_3_ that secures the absence of singularities in the solutions is slightly larger for *β*_2_ > 0 (the normal-GVD sign) than for *β*_2_ < 0 (at least, in the numerical searches that we have conducted). Hence, the choice of *β*_2_ > 0 is more appropriate for producing nonsingular QP solutions. Another important argument in favor of the choice of the normal GVD is that (quasi-) periodic solutions in the case of anomalous GVD are subject to the modulational instability, which tends to split the (quasiperiodic) wave into a chain of bright solitons [68], [69], thus making the scheme inappropriate for the experimental realization, while this instability is typically absent in the case of the normal GVD.

**Figure 1:**
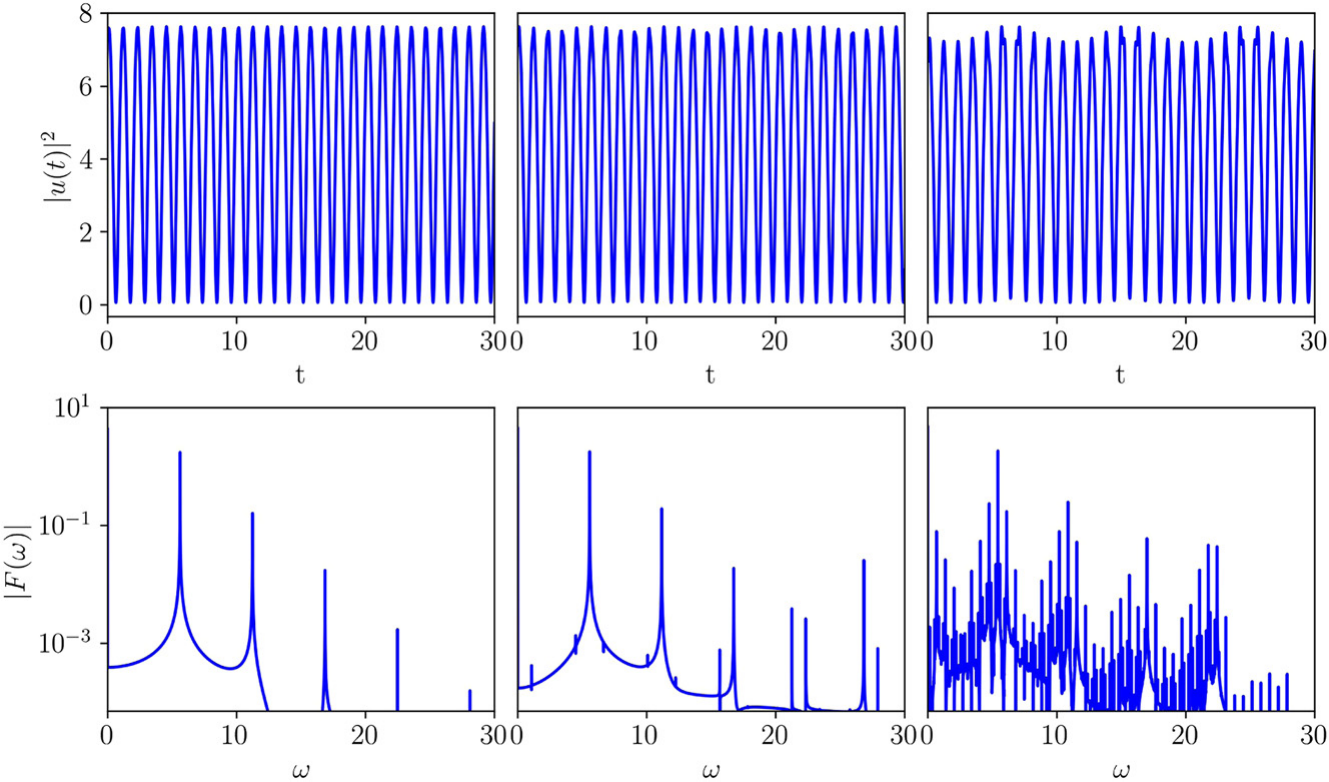
Top panels show the effective supporting potentials, |*u*(*t*)|^2^, produced by the numerical solution of Eq. (4) with initial conditions *u*(*t* = 0) = 2.5 + *i*, d*u*/d*t*(*t* = 0) = d^2^ *u*/d*t* ^2^(*t* = 0) = 1 + *i*, and parameters *β*_2_ = 1, *k* = 10. The third-order GVD coefficient takes values *β*_3_ = 0.05, *β*_3_ = 0.1, and *β*_3_ = 0.15 in the left, central, and right columns, respectively. Bottom panels show the respective spectra |*F*(*ω*)| on the logarithmic scale, see Eq. (18).

Figure 1 shows typical examples of solutions for the optical power in the support channel, |*u*(*t*)|^2^, along with their Fourier transforms, which are defined in the usual form,

(18) $$F\left(\omega \right)=\overset{+\infty }{\underset{-\infty }{\int }}{\text{e}}^{-\text{i}\omega t}|u\left(t\right){|}^{2}dt,$$

(actually, the integration in Eq. (18) is performed over the temporal range in which the numerical solution was produced). For *β*_3_ ≠ 0, the system is not integrable, and the evolution of |*u*(*t*)|^2^ is not periodic for generic initial conditions. However, in the cases of *β*_3_ = 0.05 and 0.1, presented in the left and central columns of Figure 1, the quasi-periodic behavior is very weak. This is confirmed by the presence of strong peaks only at the main frequency and its higher harmonics in the spectra plots (note that the spectra are presented on the logarithmic scale). For *β*_3_ = 0.15, shown in the right column of the figure, a well-pronounced quasi-periodic pattern emerges. This pattern is used throughout the rest of the paper to clearly demonstrate the AA localization in the signal channel.

It is relevant to stress that the numerical solution of Eq. (4) corroborates the conservation of the dynamical invariants (12) and (16), as predicted by the above analysis.

### 3.2 The Aubry–André-like localization of the signal wave in the temporal domain

Here, we move on to the core part of the work, which demonstrates the temporal localization of the signal field Ψ governed by Eq. (2) under the action of the QP supporting structure |*U*(*z*, *t*)|^2^ ≡ |*u*(*t*)|^2^ analyzed in the previous section. To this end, we look for solutions to Eq. (2) with a real propagation constant *E*,

(19) $$\Psi \left(z,t\right)=e^{i\mathrm{Ez}}\psi \left(t\right)$$

(cf. Eq. (3)), reducing the propagation equation (2) to the ODE,

(20) $$\left[-\frac{\gamma_{2}}{2}\frac{\partial^{2}}{\partial t^{2}}-\frac{i\gamma_{3}}{6}\frac{\partial^{3}}{\partial t^{3}}+2|u\left(t\right){|}^{2}\right]\psi =E\psi .$$

This equation with *γ*_3_ = 0 is tantamount to the standard 1D linear Schrödinger equation with the temporal coordinate *t*, effective mass 1/*γ*_2_, and QP potential 2|*u*(*t*)|^2^, which can give rise to the AA-like localization. In particular, comparing Eq. (20) to the discrete (tight-binding) AA model, we conclude that parameter *γ*_2_ in Eq. (20) is proportional to the intersite tunneling rate in the AA model. As concerns the third-order GVD, represented by *β*_3_ > 0, it is crucially important for the production of the QP solutions by Eq. (4), but the similar term ∼*γ*_3_ in Eq. (20) is not a major factor for the study of the localization effect in the framework of the latter equation; therefore, it will be considered elsewhere.

Proceeding to the detailed analysis, we first consider the spectrum of eigenvalues *E* produced by numerical solution of Eq. (20) with the open (Dirichlet) boundary conditions, i.e., *ψ*(*t* = 0) = *ψ*(*t* = *T*) = 0, where *T* is much larger than the time scale over which |*u*(*t*)|^2^ varies. In this context, we discretize the time coordinate as *t*_*n*_ = *n*d*t*, with integer *n* and sufficiently small d*t*, to achieve well-converging results, and represent the left-hand side of Eq. (20) in the form of an Hermitian matrix,

(21) $$\begin{array}{ll} & \;-\frac{\gamma_{2}}{2dt^{2}}\left[\psi \left(t_{n+1}\right)-2\psi \left(t_{n}\right)+\psi \left(t_{n-1}\right)\right]\\ & \;+2|u\left(t_{n}\right){|}^{2}\psi \left(t_{n}\right)=E\psi \left(t_{n}\right), \end{array}$$

which can be diagonalized by standard procedures.

**Figure 2:**
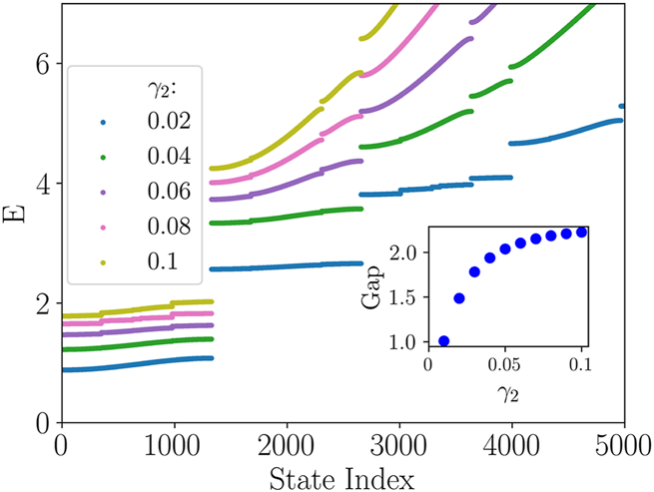
Eigenvalues *E*, in the ascending order, produced by the numerical solution of Eq. (20) with *γ*_3_ = 0 (the same is set in all figures produced below) and the effective potential 2|*u*(*t*)|^2^, which is shown in the right column of Figure 1. The solutions were obtained with the open boundary conditions, i.e., *ψ*(=0) = *ψ*(*t* = *T* ≡ 1,000) = 0. Values of *γ*_2_ are indicated in the panels. In all cases, the band structure is evident. The inset shows the gap between the first and second bands as a function of *γ*_2_.

**Figure 3:**
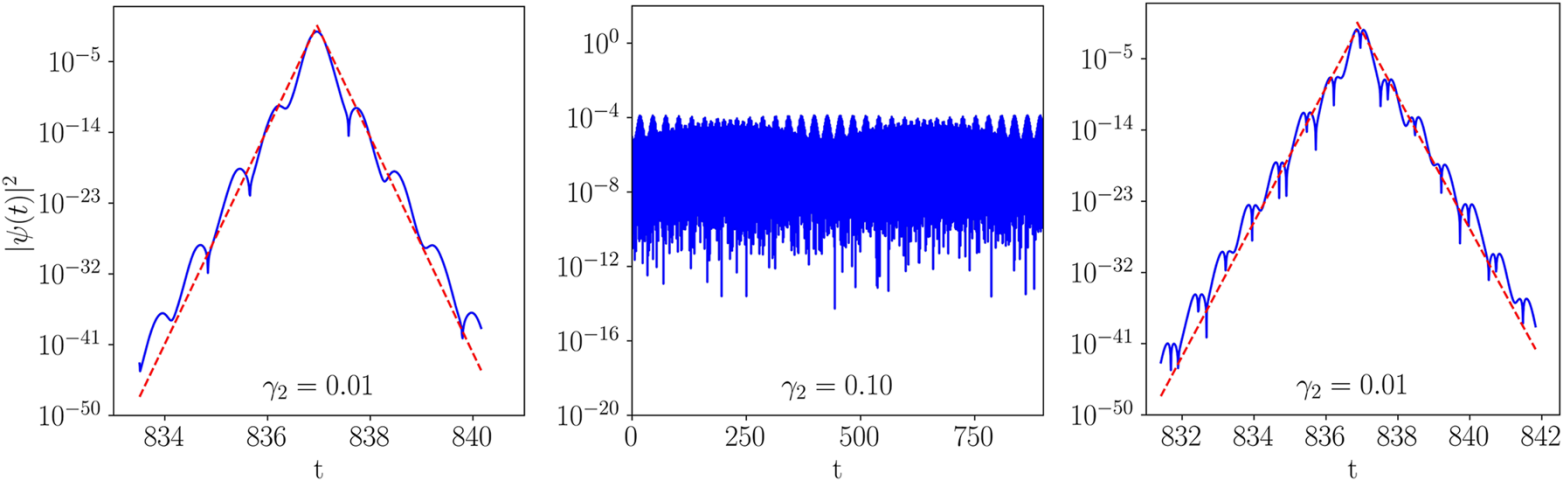
The left panel shows ${\left|\psi \left(t\right)\right|}^{2}$ , on the logarithmic scale, for the well-localized eigenstate of Eq. (20) corresponding to eigenvalue *E* in the middle of the first band for *γ*_2_ = 0.01, see Figure 2. Similarly, the middle panel shows the eigenstate for *γ*_2_ = 0.1, where no signature of the localization is observed. The right panel presents the eigenstate corresponding to eigenvalue *E* in the middle of the second band for *γ*_2_ = 0.01 (the same as in the left panel). For the same *γ*_2_ = 0.1 as in the middle panel, the respective eigenstate shows no localization signature either in the second band. In the left and right panels, we also plot exponential profiles $B\exp\left(-|t-t_{0}|/\xi \right)$ with parameters fitted to the plots of the localized eigenstates. The so obtained values of parameter *ξ* agree very well with the localization time calculated with the help of the transfer-matrix approach. Namely, the fitted values are *ξ* = 0.0752 and 0.1183 for the left and right panels, while the corresponding transfer-matrix results are 0.0727 and 0.1175, respectively. All other parameters are the same as in Figure 2.

Figure 2 shows that the eigenvalues form a band structure with gaps that become larger as *γ*_2_ increases. Examples of eigenstates from the first and second bands are shown in Figure 3. For sufficiently small values of *γ*_2_ (i.e., relatively large values of the effective mass in Eq. (20)), the QP-shaped effective potential, 2|*u*(*t*)|^2^, indeed produces exponentially confined eigenstates *ψ*(*t*), which precisely exhibit the AA localization in the temporal domain. By positioning an optical detector at some fixed location *z*, one will observe that the temporarily localized signal *ψ*(*t*) gradually emerges from zero and then exponentially decays at large times.

The localized eigenstates produced by the numerical solution of Eq. (20) decay exponentially. This feature is clearly corroborated, in the left and right panels of Figure 3, by the fitted exponential profiles, $B\exp\left(-|t-t_{0}|/\xi \right)$ . The localization time *ξ* (the counterpart of the localization length in the spatial domain) can be determined using the transfer-matrix approach [70]. To this end, we use the discrete representation of functions, *ψ*_*n*_ = *ψ*(*n*d*t*), similarly to the numerical diagonalization of Eq. (20). Then, using Eq. (20), we compute

(22) $$R_{n}\equiv \frac{\psi_{n}}{\psi_{n-1}}=\frac{2{\left(dt\right)}^{2}}{\gamma_{2}}\left(2|u_{n}{|}^{2}-E\right)+2-\frac{1}{R_{n-1}},$$

where the discretized support structure is |*u*_*n*_|^2^ = |*u*(*n*d*t*)|^2^, and we iterate *R*_*n*_ as per Eq. (22), starting from *R*_1_ ≠ 0. The localization time is thus obtained as $\xi =\lim_{N\to \infty }\left[Ndt{\left(\sum_{n=1}^{N}\log|R_{n}|\right)}^{-1}\right]$ [70]. The numerically calculated localization times closely match the values obtained from the fitting of the exponential profiles, see Figure 3.

**Figure 4:**
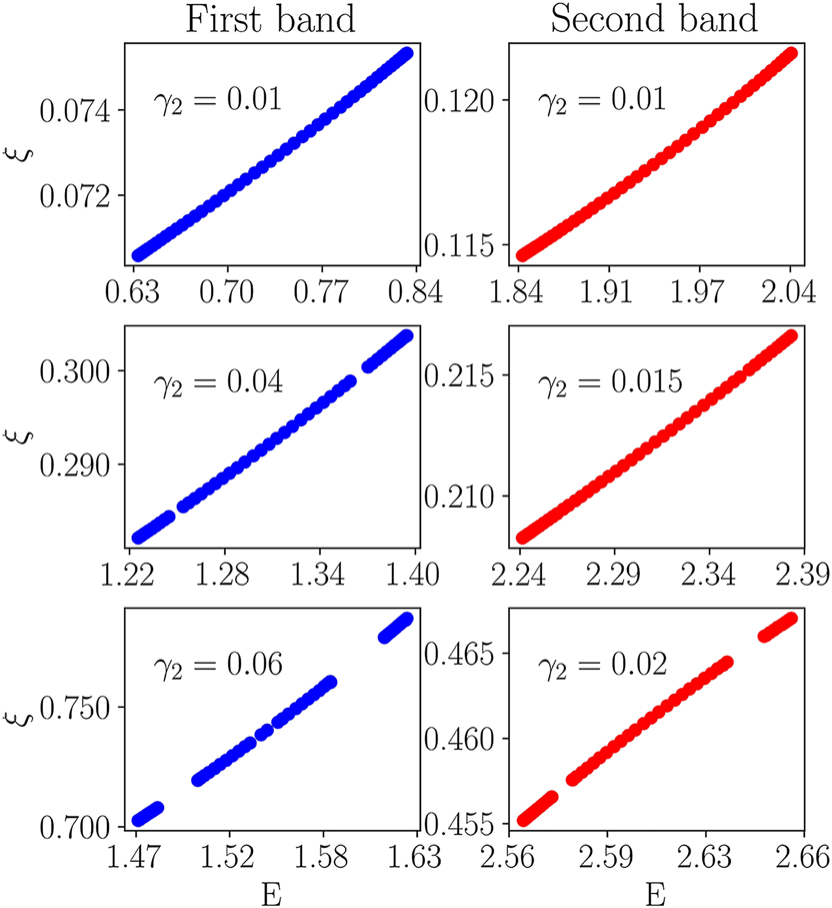
Localization time *ξ* versus eigenvalue *E*, as produced by Eq. (20), using the transfer-matrix calculations (see Eq. (22)) in the first and second bands (the left and right columns, respectively), for different values of *γ*_2_, as indicated in the panels. For certain ranges of *E*, small gaps in the eigenvalues are present within the bands (cf. Figure 2). In these gaps, the localization time is not provided (no points on the plots) because there are no solutions corresponding to these eigenvalue ranges. Other parameters are the same as in Figure 2.

**Figure 5:**
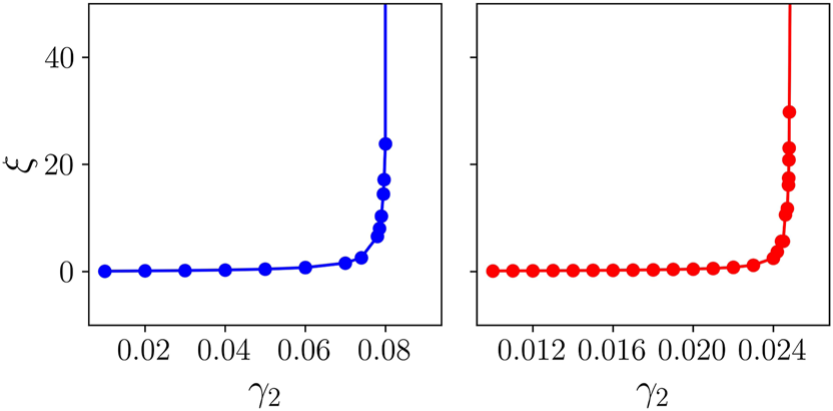
Localization time *ξ* versus the GVD coefficient *γ*_2_ in the first and second bands (the left and right panels, respectively). States from the middle of the bands were chosen, but similar behavior is observed in the entire bands. All other parameters are the same as in Figure 2.

To summarize the results produced by the systematic analysis of the present system, Figure 4 shows the localization time *ξ*, identified by means of the transfer-matrix method in the first and second bands at different values of *γ*_2_. It is seen that, in both bands, *ξ* changes slightly, being weakly sensitive to the variation of eigenvalue *E*. On the other hand, Figure 5 exhibits a steep change of *ξ* following the variation of the GVD coefficient *γ*_2_. The figure reveals that, in both bands, there is a critical value of *γ*_2_, above which the numerically obtained localization time *ξ* diverges, i.e., the localization phenomenon vanishes. This happens when the trend to spreading (delocalization) of the wave function *ψ*(*t*) becomes too strong with the growth of coefficient *γ*_2_ in Eq. (20). Such critical behavior is qualitatively similar to that in the AA model.

## 4 Conclusions

The aim of this work is to propose a new physical setup for the realization of a particular manifestation of the time-crystal behavior, *viz*., temporal localization of the Aubry–André type of linear signals in optical fibers under the action of an effective QP (quasiperiodic) potential, which is induced through the XPM (cross-phase-modulation) effect by a nonlinear QP wave copropagating in the support channel in the same fiber. The QP waves are possible when the third-order GVD (group-velocity dispersion) is taken into regard in the support channel. In particular, two dynamical invariants of the six-dimensional dynamical system governing the wave shape in the support channel are found in the exact form. The QP patterns in the support channel and solutions for the temporarily localized optical pulses in the signal channel are produced in the numerical form. Properties of the localized pulses and the boundary of their delocalization are investigated in detail.

The possibility of the experimental realization of the scheme in fiber optics is supported by essentially the same analysis of the physical setting which was presented in terms of the two-channel system in Refs. [62] and [63]. The temporal localization of the signal pulses reported in the present work may find an application to fiber-optic communications, as it offers a possibility to maintain the *return-to-zero* regime of the data-stream transmission [60] in the low-power linear regime. As an extension of the work, it may be interesting to consider effects of nonlinearity and third-order GVD in the signal channel. It is expected that, depending on the sign of GVD in this channel, the nonlinearity may enhance the localization (leading to the formation of bright solitons [60]) or stimulate delocalization [2].
